# The Impact of Contextual, Maternal and Prenatal Factors on Receptive Language in a Chilean Longitudinal Birth Cohort

**DOI:** 10.1007/s10578-020-01091-5

**Published:** 2020-11-01

**Authors:** María Francisca Morales, Chamarrita Farkas, Eleanor Aristotelous, Angus MacBeth

**Affiliations:** 1 School of Health in Social Science, The University of Edinburgh, Edinburgh, UK; 2 Escuela de Psicología, Pontificia Universidad Católica de Chile, Santiago, Chile

**Keywords:** Resource access, Maternal characteristics, Prenatal factors, Social support, Receptive language

## Abstract

A secondary analysis was conducted on longitudinal data collected from ELPI, a representative Chilean survey to model Chilean infant’s receptive language using contextual, maternal and prenatal factors. The sample for the current study comprised children aged between 36 and 48 months (n = 3921). The sample was re-assessed when children were aged 60–72 months (n = 3100). Linear regression analyses were conducted. At the first time point, all the predictors included were significant (living area, health system provision, maternal intelligence and education, adolescent pregnancy, maternal medical appointments during pregnancy, and presence of a significant other at childbirth), except for smoking during pregnancy. The model explained 13% of the variance. However, when timepoint one receptive language scores were included in the analyses for when children were aged 60–72 months, only two variables remained as significant predictors: previous receptive language scores and maternal education, explaining 21% of the variance. Findings and implications are discussed.

**Electronic supplementary material:**

The online version of this article (10.1007/s10578-020-01091-5) contains supplementary material, which is available to authorized users.

## Introduction

Communication and language skills are complex cognitive and social features that develop across the lifespan. However, the first years of life are especially crucial as they provide the foundation for the emergence of receptive and expressive language, which in turn facilitates communication across multiple social contexts [1]. Receptive language relates to the process in which signals are understood, that is, the capacity to comprehend what is spoken, written, or signed by others [2]. Evidence suggests that higher receptive language functioning facilitates emotional regulation, promotes social and cognitive development [3]; enhances behavioural inhibition [4]; and improves executive functioning [4]. Higher receptive language skills therefore confer considerable advantages on children’s development with implications for socio-emotional abilities, academic achievement, and school adjustment [5]. Consequently, this has important cross-overs into early childhood development policies and interventions for at-risk groups.

Evidence suggests that environmental and demographic factors have a central function in children’s receptive language development [6]. In this sense, language performance in infants shows the impact of several distal and proximal factors such as the broader social context and social interactions with their primary caregivers [7]. Consequently, maternal, family and contextual characteristics have been a central framework for the study of receptive language development [8].

One factor previously considered is the social context in which the mother and the child live. In this regard, socioeconomic status (SES) has been shown to be essential for language development [9]. For instance, longitudinal research by Hart and Risley [10] found that on average, high-SES children heard approximately 2000 words, while children of low-SES heard about 500 words per hour, stating that socioeconomic factors influence the quantity and quality of maternal vocal interactions. Additionally, Lopez Boo [11] found that risk factors, such as uncertainty of income and problems accessing health and educational resources, lead to increased parental stress. SES, therefore, has an impact on children’s language development through factors such as parental features [12], cognitive stimulation [10, 13], and stress exposure [11].

A second key feature is the impact of maternal characteristics. There is consistent evidence demonstrating the importance of the mother's intelligence, educational level, and age [14, 15]. Maternal education has indirect effects on development via shaping parenting practices and children’s home settings [16]. For example, mothers with higher educational levels and literacy abilities communicate differently with their children than mothers with lower levels of education and language skills [17]. Additionally, maternal IQ is associated with children's development, impacting language outcomes directly and also indirectly through family income and home environment [18]. Maternal age is also related to child outcomes [19]. For instance, children of adolescent parents are at higher risk of struggling with emotional and cognitive problems than children of adult parents [20]; and adolescent mothers may be less verbally sensitive and more intrusive than older mothers [21].

There is also considerable research highlighting that exposure to risk factors during pregnancy, including antenatal smoking [12], perinatal depression [22] and maternal anxiety and stress [23], impacts upon subsequent offspring development [24]. There is growing evidence that undiagnosed antenatal depression is the leading risk factor for postnatal depression [25, 26]. Due to the confounding of common somatic depressive symptoms (e.g. sleep, energy, appetite) as typical pregnancy signs [27], depression is often undetected. Antenatal and postpartum depression have a negative effect on children’s language outcomes [28], both directly and via increased risk of adverse parenting practices such as low maternal sensitivity, and negative parent–child interactions [5, 29]. However, evidence from longitudinal studies has been equivocal regarding the long-term effects of maternal depressive symptoms on children’s language performance [30–32].

Finally, maternal social support may also mediate associations between maternal characteristics and language development [33]. The adverse effects of risk factors such as maternal depression or adolescent pregnancy can be reduced if the mother has adequate social support from her family, friends and professionals [31, 34].

Although ample evidence exists regarding contextual, maternal and prenatal characteristics, most research has been carried out in developed countries [35]. By contrast, in regions transitioning from developing to developed countries, especially Latin American states, there have been fewer studies on children’s development [36]. Furthermore, developing countries have lower rates of school achievement, higher illiteracy levels, and worse standards in international language tests than developed states [37]. Therefore, while significant progress has been made in reducing child mortality and malnutrition in most countries within this region, progress in other dimensions of child development such as language remains insufficient [38]. Nevertheless, interest in promoting child development in the region has grown, and several governments have included this subject in their policy agenda [38]. Additionally, three of these countries (Chile, Colombia, and Uruguay) have developed large cohort studies to increase research on children’s development [39].

Chile has demonstrated strong economic progress and is transitioning to developed country status. However, based on OECD Gini coefficients, Chile also manifests significant levels of inequality across the population [40]. Social inequality in Chile has clear consequences for early child development with children under five years old from low-SES backgrounds presenting significantly elevated rates of cognitive and language delays compared to children from high-SES backgrounds [39]. Regarding language development, Chile's results in international cognitive and language assessment tests (PISA) are above the average of other Latin American states [41], but significantly below the standard of developed countries [42]. Furthermore, comparing language delay in children internationally suggest that 28.3% of children in Chile under five years old achieved lower scores than expected on receptive language [43] compared with 10% of children in the USA [44]. There are also in-country geographical differences with reports that 37.5% of preschool children in southern Chile exhibited receptive language delay [45].

Small scale research on predictors of language development in Chile identifies socioeconomic circumstances and maternal educational level [46, 47] as key predictors. Regarding the impact of SES, Farkas and Corthorn [48] reported that, at 12 months old, there is a relationship between language development and SES where children of higher SES use a more productive vocabulary than children of a medium and low SES; and these differences are still evident at 30 months old [47]. Additionally, children living in rural sectors achieved lower language scores on standardised national tests than children living in urban zones, a result that is explained as a consequence of socioeconomic deprivation in rural areas [49]. Mothers from low-SES backgrounds in Chile also exhibit higher levels of parental stress and more mental health problems, as a result of contextual adversity [50]. Moreover, regarding maternal education, Chilean research has shown that better receptive language outcomes are strongly associated with higher maternal education levels [34, 46]. This relationship can be explained by (i) the association between structural lifestyle conditions and different educational levels and (ii) by the quality of mother–child interaction, e.g. the extent to which mothers engage in stimulating linguistic activities with their children [46]. However, these findings have all been limited by small sample sizes, use of only one or two geographical locations in Chile, and cross-sectional designs.

Therefore, although there is evidence in Chile for the impact of resource access, maternal, and prenatal features on children’s language development over time, there has thus far been no evidence from a large-scale population representative cohort. The current study addresses this by using data of ELPI (Encuesta Longitudinal de la Primera Infancia: Longitudinal Survey of Early Infancy), a large representative survey of Chilean children. Specifically, we asked the following research questions: First, are resource access, maternal characteristics, prenatal risk factors, and perinatal social support associated with receptive language in Chilean children aged between 36 and 48 months old? Second, do these associations remain stable after two years when children are aged between 60 and 72 months old?

The first age range, 36–48 months old, was selected as children will have already achieved greater complexity in different aspects of language development [1]; and to ensure consistency with similar international research assessing receptive language [44, 51]. It was hypothesised that better receptive language development in children would be associated with higher maternal SES, educational and intelligence levels, absence of prenatal risk factors, and better perinatal social support.

## Method

### Design and Data Source

This study was a secondary analysis of longitudinal data collected from ELPI. This is a representative family cohort from all regions of Chile conducted between 2010 and 2012. In the first timepoint of ELPI (2010) children were between seven months to five years old (*n* = 15,175); in the second timepoint (2012) they were aged between fifty-four months to seven years old [52]. Full details of the ELPI recruitment and cohort design are available elsewhere [46]. Children’s receptive language scores were described and compared considering the categories for the different factors, and these factors were analysed for their predictive value on children’s receptive language at both timepoints.

### Participants

The present research considered a sub-sample of the ELPI. The inclusion criteria for this sample were children aged between 36 and 48 months in 2010 (*n* = 4013, 26.5% of the total ELPI cohort). The exclusion criteria were: children not living with their biological mother (*n* = 68); and those with no receptive language measurement (*n* = 24), leaving a final sample of 3921 cases (25.8% of the total ELPI cohort). The final sample included 51.6% of families living in the central area of the country, 13.6% in the north and 34.8% in the south. For maternal age, 25.9% of mothers were between 18 to 24 years old, 44.5% between 25 to 34 years old, and 29.3% of mothers had more than 35 years old. All the mothers spoke Spanish with their children during daily activities. Regarding children’s gender, 49.3% of them were female, and 50.7% were male. The majority of children (64.7%) were attending pre-school. Demographic characteristics of the sample are presented in Table 1. Attrition analyses were performed comparing the entire cohort to those cases included in the current sample, showing that only the area of residence presented a significant difference (see online supplement 1).

**Table 1:** Characterisation of the sample considering the predictors

	N	%
Area of residence		
Urban	3503	89.3
Rural	418	10.7
Health provisional system		
Public system	3446	89.7
Private system	397	10.3
Maternal educational level		
No formal education	17	0.4
Primary complete	704	18.1
Secondary complete	1578	40.6
Vocational training	1163	30.0
University studies	398	10.2
Postgraduate studies	23	0.6
Maternal IQ (WAIS)		
Digit span subtest		
Below average	2623	66.9
Average or high	1298	33.1
Vocabulary subtest		
Below average	1352	34.5
Average or high	2569	65.5
Adolescent pregnancy		
Yes	812	20.7
No	3109	79.3
Prenatal depression		
Yes	377	9.9
No	3441	90.1
Smoking cigarettes at pregnancy		
Yes	364	9.3
No	3554	90.7
Med. Appointments pregnancy		
Below recommendation	490	12.6
According recommendation	3389	87.4
Mother accompanied at childbirth		
Yes	2831	72.3
No	1085	27.7
Postnatal depression		
Yes	416	10.7
No	3468	89.3
Receptive language	Min–Max	M (SD)
Time point 1	69–145	103.82 (16.25)
Time point 1	55–145	105.98 (19.08)

N = 3921

At the second timepoint (2012), 20.9% of the cases were missing, leaving n = 3100 cases. Attrition analyses were repeated between children that had receptive language measurement at both timepoints and children that only had measures in 2010. No significant baseline differences emerged between groups (see online supplement 2).

### Measures and Variables

#### Receptive Language

Children’s language outcomes were assessed using the Spanish version of the Peabody Picture Vocabulary Test (TVIP), 3rd version [53]. The TVIP is a widely used measure of receptive vocabulary abilities for children aged 30 months and older. In the test, children are shown four images and asked to find the picture that corresponds to the stimulus word named by the administrator. The TVIP has been validated for use in Puerto Rican and Mexican communities and adjusted for Chilean language (uncommon words were replaced with more frequent terms), showing very high internal consistency (K-R = 0.98) with a correlation of 0.95 between the two versions [54]. For this study standardised receptive scores were used. The TVIP was conducted during 2010, when the children were between 36 and 48 months old (M = 42 months, SD = 3.69). The assessment was repeated in 2012, when the children were between 60 and 72 months old (M = 68 months, SD = 4.12).

#### Language Predictors

##### Contextual Factors

This information was collected from the first wave of the study, including information on the area of residence (urban or rural) and type of health provision system (private or public). The health provisional system was considered a substitute for access to economic resources as the private system in Chile is associated with a higher economic status and the public system with a lower family income [55].

##### Maternal Characteristics

Data from the first wave was used to gather information on maternal educational level (no formal education, primary, secondary, vocational, university, or postgraduate studies). Maternal intelligence was assessed using the Wechsler Intelligence Adult Scale 3rd version (WAIS-III) [56]. This test measures adult intellectual performance through 12 subtests; however, only the vocabulary and digit span subtests were considered in ELPI. This test was adjusted for the Chilean population yielding good reliability and validity scores [57, 58]. For this study, both subtests were used as a dichotomous variable with a below-average category (standardised score less than 8) and an average/high category (standardised score of 8 and up).

##### Prenatal Risk Factors

These variables were collected from the interview in the first wave. Four variables were considered as prenatal risk factors: prenatal depression (yes/no); smoked cigarettes at pregnancy (yes/no); if it was an adolescent pregnancy (yes/no); and postnatal depression (yes/no).

##### Perinatal Social Support

Finally, two variables were added as indicators of perinatal social support: the number of doctor appointments during pregnancy stratified according to the Chilean early childhood national program recommendation of seven or more appointments (below the recommendation/ according to the recommendation) [59]; and whether the mother was with a significant person during childbirth (yes/no).

### Data Analysis

Statistical analyses were performed using *Statistical Package for the Social Sciences SPSS® 23*. First, missing data between variables and timepoints was conservatively controlled using the list-wise method. Descriptive statistics were obtained for receptive language scores, and then ANOVA analyses were performed to detect significant differences between categories using dummy variables. Finally, after checking the classic assumptions, linear regression analyses were conducted in both timepoints using the "hierarchical" method, where a fixed order for entering the predictor variables was specified in order to test the independent effects of each predictor. The regression model was constructed with four blocks of variables: 1. Resource access; 2. Maternal characteristics; 3. Prenatal risk factors; and 4. Perinatal social support.

### Ethics

Ethical evaluation of ELPI was conducted by the University of Chile. The current analyses received ethical approval from the University of Edinburgh School of Health and Social Science Research Ethics Committee.

## Results

### Receptive Language Predictors when Children Aged 36–48-Month-Old

The receptive language average score for children between 36 and 48 months old was 103.82 (SD = 16.25). Based on bivariate analyses, children with higher receptive language scores were more likely to have mothers living in urban areas, attend the private health system, with higher maternal IQ scores and educational levels, not have had an adolescent pregnancy or have smoked in this period, and who reported that they attended all the recommended medical appointments during pregnancy and who were accompanied by a significant person during childbirth.

For maternal depression indicators (pre- and postnatal depression), results showed no significant differences in children’s receptive language scores of mothers who did report a depression diagnosis in comparison to those who did not. Table 2 shows descriptive statistics and bivariate analyses of receptive language scores for each predictor.

**Table 2:** Descriptive statistics and bivariate analyses of receptive language in the first timepoint

Variable	n	M	SD	Min–Max	ANOVA
Total sample	3921	103.82	16.25	69–145	
Resource access					
Area of residence					F(1,3919) = 48.555, *p* = 0.000
Urban	3232	104.48	16.17	69–145	
Rural	397	98.43	15.41	69–145	
Health provisional system					F(1,3841) = 142.839, *p* = 0.000
Public system	3248	102.78	15.84	69–145	
Private system	381	112.74	16.52	69–145	
Maternal characteristics					
WAIS digit span subtest					F(1,3919) = 141.227, *p* = 0.000
Below average	2441	101.70	15.58	69–145	
Average or high	1188	108.19	16.57	69–145	
WAIS vocabulary subtest					F(1,3919) = 251.051, *p* = 0.000
Below average	1239	98.17	14.65	69–145	
Average or high	2390	106.75	16.19	69–145	
Maternal educational level					F(5,3877) = 67.722, *p* = 0.000
No formal education	16	96.31	12.6	81–125	
Primary complete	661	96.78	14.6	69–145	
Secondary complete	1472	102.85	15.52	69–145	
Vocational training	1089	106.07	16.11	69–145	
University studies	370	113.08	15.33	75–145	
Postgraduate studies	21	119.81	20.27	73–145	
Pretnatal risk factors					
Prenatal depression					F(1,3816) = 1.471, *p* = 0.225
Yes	355	102.91	15.13	73–145	
No	3274	103.92	16.31	69–145	
Smoking at pregnancy					F(1,3916) = 4.481, *p* = 0.034
Yes	315	102.06	15.31	72–145	
No	3314	103.99	16.28	69–145	
Adolescent pregnancy					F(1,3919) = 14.391, *p* = 0.000
Yes	745	101.62	14.89	69–145	
No	2884	104.39	16.48	69–145	
Postnatal depression					F(1,3882) = 3.028, *p* = 0.082
Yes	372	105.28	16.29	69–145	
No	3257	103.66	16.18	69–145	
Perinatal social support					
Med. appointments pregnancy					F(1,3877) = 10.008, *p* = 0.002
Below recom	461	101.64	16.34	69–145	
According recom	3168	104.14	16.16	69–145	
Accomp. at childbirth					F(1,3914) = 95.061, *p* = 0.000
Yes	2623	105.34	16.3	69–145	
No	1006	99.86	15.26	69–145	

Multivariate analyses were conducted using a parsimonious approach. Therefore, variables not significant at a bivariate level (pre and postnatal depression) were not considered for the regression analyses. The remaining variables met assumptions for linearity, homoscedasticity, no multicollinearity, independence of error terms, and normality. Next, a linear regression was conducted using the "hierarchical" method (see Table 3), following the subsequent blocks. The first model (1) entered resource access, the second (2) maternal characteristics, the third (3) prenatal risk factors, and the final one (4) perinatal social support.

**Table 3:** Predictors of receptive language at 36 to 48 months old

	Model 1		Model 2		Model 3		Model 4	
Predictors	β	Sig	β	Sig	β	Sig	β	Sig
Constant		0.000		0.000		0.000		0.000
Resource access								
Area (urban)	0.10	0.000	0.06	0.000	0.06	0.000	0.05	0.001
Health system (public)	− 0.18	0.000	− 0.09	0.000	− 0.09	0.000	− 0.08	0.000
Maternal characteristics								
Digit span subtest (below ave.)			− 0.10	0.000	− 0.10	0.000	− 0.10	0.000
Vocabulary subtest (below ave.)			− 0.15	0.000	− 0.15	0.000	− 0.15	0.000
Maternal education			0.17	0.000	0.17	0.000	0.16	0.000
Prenatal risk factors								
Smoking at pregnancy (y)					− 0.01	0.380	− 0.01	0.408
Adolescent pregnancy (y)					− 0.04	0.020	− 0.04	0.020
Perinatal social support								
Medical appoint. (recom.)							0.03	0.043
Accomp. at childbirth (y)							0.07	0.000
Model summary	Model 1		Model 2		Model 3		Model 4	
F	89.85**		110.09**		79.63**		64.60**	
R^2^ adjusted	0.05		0.13		0.13		0.13	

*p < 0.05, **p < 0.01. N = 3921

The first model indicated that both resource access variables were significant predictors of children’s receptive language, showing that living in an urban area had a positive relationship with children’s receptive language scores (*β* = 0.10, *t* = 6.06, *p* ≤ 0.001), and being part of the public health system a negative one (*β* =  − 0.18, *t* =  − 11.38, *p* ≤ 0.001). In model 2, controlling for maternal characteristics, the results indicated that both intelligence subtests (digit span and vocabulary) were significant predictors, with mothers with poor scores in both intelligence subtests having children with lower achievements in receptive language (digit span: *β* =  − 0.10, *t* =  − 6.05, *p* ≤ 0.001; vocabulary: *β* =  − 0.15, *t* =  − 9.39, *p* ≤ 0.001). Moreover, it is shown that maternal education was a positive predictor of children’s language scores (*β* = 0.17, *t* = 9.76, *p* ≤ 0.001).

In Model 3 only the adolescent pregnancy variable emerged as a significant predictor within the prenatal risk factors, with a negative association with children’s receptive language (*β* =  − 0.04, *t* =  − 2.32, *p* = 0.020). Finally, both perinatal social support variables were significant predictors, indicating that mothers who went to the recommended medical appointments during pregnancy (*β* = 0.03, *t* = 2.02, *p* = 0.043) and who were with a significant person at childbirth (*β* = 0.07, *t* = 4.07, *p* ≤ 0.001) have children with better receptive language scores. The final model was significant (*F* = 64.60, *p* ≤ 0.001), with eight statistically significant predictors explaining 13% of the variance in receptive language.

### Receptive Language Predictors when Children Aged 60–72-Month-Old

When children were between 60 and 72 months old, their receptive average score was 105.98 (SD = 19.08), showing an increase in comparison to the first timepoint results (2010: M = 103.82; SD = 16.25). Timepoint 1 and 2 scores were significantly correlated (*r* = 0.43; *p* ≤ 0.001). A paired sample t-test analysis showed a significant difference between both periods: (*t* (3099) =  − 5.526, *p* ≤ 0.001). Therefore, in the second timepoint analyses, previous receptive language performance was included as a new variable in the final regression model.

For the bivariate analyses, children with higher receptive language scores had a higher frequency of mothers living in urban areas, using the private health system, with better IQ scores and educational levels, without having reported an adolescent pregnancy, and who were accompanied by a significant person during childbirth. In contrast to the first timepoint, at this stage, smoking during pregnancy and not attending all recommended medical appointments during pregnancy were not significant in comparison to those mothers who did not smoke and who went to all the recommended medical appointments at pregnancy. Prenatal risk factors that were not significant at the first timepoint (pre- and postnatal depression) continued to have no significant effect on children’s receptive language scores. Table 4 shows descriptive statistics and bivariate analyses of receptive language scores for each predictor.

**Table 4:** Descriptive statistics and bivariate analyses of receptive language in the second timepoint

Variable	n	M	SD	Min- Max	ANOVA
Total sample	3100	105.98	19.08	55–145	
Resource access					
Area of residence					F(1,3098) = 24.488, *p* = 0.000
Urban	2548	106.49	18.82	55–145	
Rural	326	101.26	20.18	55–143	
Health provisional system					F(1,3037) = 45,455, *p* = 0.000
Public system	2597	105.17	19.11	55–145	
Private system	277	112.73	17.01	55–145	
Maternal characteristics					
WAIS digit span subtest					F(1,3098) = 49.039, *p* = 0.000
Below average	1970	104.34	19.06	55–145	
Average or high	904	109.29	18.59	55–145	
WAIS vocabulary subtest					F(1,3098) = 86.945, *p* = 0.000
Below average	1002	101.58	18.77	55–145	
Average or high	1872	108.21	18.79	55–145	
Maternal educational level					F(5,3065) = 43.738, *p* = 0.000
No formal education	13	94.00	27.19	55–145	
Primary complete	536	98.05	19.11	55–145	
Secondary complete	1192	105.22	18.64	55–145	
Vocational training	855	109.02	18.09	55–145	
University studies	263	114.19	16.76	55–145	
Postgraduate studies	15	126.87	12.65	103–145	
Pretnatal risk factors					
Prenatal depression					F(1,3016) = 1.664, *p* = 0.197
Yes	267	104.58	19.72	55–145	
No	2607	106.03	18.97	55–145	
Smoking s at pregnancy					F(1,3095) = 1.965, *p* = 0.161
Yes	245	104.52	16.96	55–145	
No	2629	106.02	19.23	55–145	
Adolescent pregnancy					F(1,3098) = 6.185, *p* = 0.013
Yes	575	104.20	18.28	55–145	
No	2299	106.32	19.21	55–145	
Postnatal depression					F(1,3067) = .581, *p* = 0.446
Yes	285	106.31	19.83	55–145	
No	2589	105.85	18.96	55–145	
Perinatal social support					
Med. appointments pregnancy					F(1,3069) = .986, *p* = 0.321
Below recom	363	105.14	18.84	55–145	
According recom	2511	106.01	19.08	55–145	
Accomp. at childbirth					F(1,3095) = 37.523, *p* = 0.000
Yes	2051	107.24	18.87	55–145	
No	823	102.54	19.08	55–145	

Again, following the principle of parsimony, the subsequent variables were not included for multivariate analyses: prenatal depression, smoking cigarettes during pregnancy, postnatal depression and medical appointments in pregnancy in accordance with the Chilean recommendation. Linear regression was conducted using the "hierarchical" method (see Table 5), with the following blocks: the first model (1) entered resource access, the second (2) maternal characteristics, the third (3) prenatal risk factors and perinatal social support, and the final one (4) previous receptive language scores.

**Table 5:** Predictors of receptive language at 60 to 72 months old

	Model 1		Model 2		Model 3		Model 4	
Predictors	β	Sig	β	Sig	β	Sig	β	Sig
Constant		0.000		0.000		0.000		0.000
Resource access								
Area (Urban)	0.08	0.000	0.04	0.014	0.04	0.029	0.02	0.236
Health system (Public)	− 0.12	0.000	− 0.04	0.028	− 0.03	0.064	0.00	0.932
Maternal characteristics								
Digit span subtest (below ave.)			− 0.05	0.005	− 0.05	0.005	− 0.02	0.259
Vocabulary subtest (below ave.)			− 0.09	0.000	− 0.09	0.000	− 0.03	0.136
Maternal Education			0.20	0.000	0.19	0.000	0.13	0.000
Prenatal risk factors and perinatal social support								
Adolescent pregnancy (y)					− 0.03	0.121	− 0.02	0.259
Accomp. at childbirth (y)					0.04	0.041	0.01	0.456
TVIP score 2010							0.38	0.000
Model summary	Model 1		Model 2		Model 3		Model 4	
F	32.85		53.26		39.01		98.72	
R^2^ adjusted	0.02**		0.08**		0.08**		0.21**	

*p < 0.05, **p < 0.01. N = 3100

The first model demonstrated that resource access variables were still significant predictors of children’s receptive language at this stage, indicating that maternal urbanicity had a positive relationship with children’s receptive language scores (*β* = 0.08, *t* = 4.44, *p* ≤ 0.001) and public health system attendance was negatively associated (*β* =  − 0.12, *t* =  − 6.41, *p* ≤ 0.001). Model 2 highlight that the intelligence subtests (digit span and vocabulary) were significant predictors of children’s receptive language, showing that mothers with lower than average scores in both intelligence subtests had children with lower results for receptive language (digit span: *β* =  − 0.05, *t* =  − 2.84, *p* = 0.005; vocabulary: *β* =  − 0.09, *t* =  − 4.92, *p* ≤ 0.001). Moreover, the maternal education variable was a positive predictor of children’s language scores (*β* = 0.20, *t* = 10.05, *p* ≤ 0.001). Model 3 indicated that when the prenatal risk factors and perinatal social support variable are considered, the health system was no longer significant (*β* =  − 0.03, *t* =  − 1.85, *p* = 0.064). Additionally, the model showed that mothers who were accompanied by a significant person at childbirth had children with better receptive language scores (*β* = 0.04, *t* = 2.05, *p* = 0.041). Adolescent pregnancy was not a significant predictor 2 years after the first time point. Finally, when model 4 included the previous receptive language achievements, only two variables emerged as significant predictors: maternal education (*β* = 0.13, *t* = 7.15, *p* ≤ 0.001) and children’s receptive language scores of 2010 (*β* = 0.38, *t* = 21.76, *p* ≤ 0.001). This final model considering these two predictors was significant (*F* = 98.72, *p* ≤ 0.001), and explained 21% of the variance in receptive language.

## Discussion

Using data from a large, nationally representative survey from Chile, this study analysed predictors of receptive language in children aged between 36 and 48 months old. It then analysed whether these associations persisted after two years, integrating receptive language scores for the first timepoint in the final model. Regarding the first research question, findings showed that, when multiple factors are examined together, resource access, maternal characteristics, and perinatal social support emerge as significant predictors of receptive language, more so than most of the prenatal risk factors. Therefore, after social background is considered, prenatal risk events seem to play only a minimal role in language development in children aged between 36 and 48 months old. These results align with longitudinal international research which indicates that family risk variables (e.g., low income, SES, neighbourhood) are the strongest predictors of language development in kindergarten and that prenatal risk factors although related to health at birth do not have a direct relationship with language outcomes [60]. Additionally, our full model explained only a modest amount of variance in receptive language (13%), consistent with the findings of previous research that show most of the variance in receptive language at this stage is unknown, even after incorporating a substantial number of contextual and prenatal predictors [61, 62].

Maternal characteristics emerged as the strongest predictors of receptive language between 36 and 48 months old. These findings demonstrate that better maternal cognitive skills and higher educational levels predict better receptive language outcomes for children. This is consistent with international and local evidence, suggesting that maternal IQ and greater education buffer against developmental problems [63] and language difficulties [16, 18, 46]. Additionally, findings related to resource access were consistent with previous Chilean and international research [9, 11, 47], indicating that living in urban areas and being part of the private health system—which is itself strongly related to high SES in Chile [49, 55]—had a positive impact on children’s receptive language. In contrast, lower socioeconomic level and rural location may put children at higher risk for suboptimal receptive language development. Regarding prenatal risk factors, only the adolescent pregnancy variable was significant in the first timepoint. However, similar research [60] has considered this variable as a maternal characteristic rather than a prenatal feature; therefore, the findings of the first timepoint are similar to those that consider the importance of maternal characteristics over prenatal features. Finally, perinatal social support factors are important variables to consider in language development when children are 36–48 months old. These results are consistent with previous studies which found that maternal social support factors are essential features that could impact the association between maternal characteristics and children’s developmental outcomes [34].

Our results also showed that after two years, only maternal education remained as a significant predictor of child receptive language, in comparison to the eight significant variables in timepoint one. Moreover, the second timepoint results indicated that the previous receptive language development emerged as the strongest significant predictor of receptive language at this stage, explaining 21% of the variance in receptive language when children were 60–72 months old. These findings highlight the importance of prior language development, potentially supporting a critical period role for most of the predictors that were significant in the first timepoint, with their unique contribution thereafter being superseded by the child’s own receptive language acquisition. A similar study from an Australian cohort identified that the combination of child, family and maternal factors explained 18.9% of the variance in receptive language scores at 4 years of age, which was substantially higher than the variance explained in the same cohort at 2 years of age (4% of the receptive language variance) [64]. Additionally, the finding in the current study that that previous receptive language predicts later language development is consistent with meta-analytic evidence identifying the importance of previous receptive language performance for later language development [65]. Our findings thus support other similar cohort identifying commonalities between a confluence of factors, including maternal education, socioeconomic factors, and previous receptive language in predicting later language development. This points towards a number of cross-cultural longitudinal factors that form a prototypical trajectory for receptive language development, whereby some factors play a significant role during early childhood, and the impact of other factors become more important in later stages of childhood.

The current findings extend our knowledge of child development compared to evidence from previous Chilean research. Study strengths include the use of a large representative sample and a longitudinal design with the same standardised measure of receptive vocabulary. Nevertheless, results should be considered with caution due to several design and measurement limitations. First, the study evaluates the predictors of children of 36–48 and 60–72 months old, which means that the results do not generalise to children of other ages. Second, measuring language through TVIP is restricted as it provides a limited evaluation of language development by focusing only on receptive language: it does not assess other language areas such as vocabulary or expressive skills. Additionally, the measurement was originally validated in Puerto Rican and Mexican cultures and, although adapted to the Chilean population, norms are based on other populations in a different time context. Therefore, further studies in the area should include more holistic measures of infant’s language development, validated in the Chilean population, to provide a more comprehensive assessment of linguistic skills. Moreover, a holistic method to measure language could allow more sophisticated statistical analysis such as identifying latent constructs using structural equation modelling. Third, the ante- and postnatal depression variables are a limited source of information due to the use of retrospective self-reports, which are susceptible to social desirability bias [66]. Future research could include administrative data to confirm diagnoses or more extensive questions about the symptoms, context, and length of the diagnosis. Overall, most of the predictors we considered in the current study did not emerge as significant variables in the second timepoint, with only maternal education and the child’s prior language development reaching significance, with a susbstantial proportion of variance left unexplained. This indicates that there are likely to be other relevant predictors that this study did not consider and that could account for a larger variance. For example, previous studies have found significant relationships between children’s receptive language and a number of social determinants of development including pre-school attendance [61], the level of extended family support [67], and neighborhood environment [60]. Thus, future studies should consider these and other predictors. Fourth, a conservative missing data method employed in the current study means that relevant information within cases and timepoints could have been lost. Therefore, further studies should use other statistical strategies to deal with missing values, such as multiple imputation [68]. As new waves of ELPI data become available, more complex longitudinal analyses such as latent class growth modelling [69], will become available, enabling exploration of children’s language trajectories. Finally, the current study only evaluated the impact of maternal characteristics. To fully support research on parental effects upon child development [70], future research should consider the importance of additional significant caregivers, such as fathers and members of the extended family.

These limitations notwithstanding, study results contribute to the existing body of literature on language predictors, particularly in a Latin American context. These findings could therefore be used as a guideline to improve public policies targeting early childhood in Chile and Latin America. Specifically, second timepoint results emphasise that previous language performance is one of the most robust predictors of children’s receptive language at preschool stage. Thus, early interventions that promote adequate initial development comprise an effective strategy to improve future language development. Furthermore, this research highlights the importance of maternal social support. Hence, public health policies should promote the participation of mothers and other significant relatives in health, educational, and community initiatives to increase maternal social support in crucial developmental stages such as the perinatal period. Consequently, maternity programmes within health systems should encourage a significant relative to accompany mothers to medical appointments during pregnancy, at the time of childbirth, and during the first year of the baby’s life. Current Chilean programmes focusing on early childhood, such as *Chile Crece Contigo* (Chile Grows with you), could identify specific social groups who require more initial support addressing specific risk factors. For instance, programmes could be adapted for mothers who did not finish basic education, have lower cognitive abilities, families who are living in disadvantaged economic conditions, and rural families with resource access difficulties.

### Summary

The first years of life are crucial to acquire the foundations for receptive language, which in turn impacts children’s developmental outcomes. The study of language predictors highlights the importance of considering as relevant factors the social environment, maternal characteristics and prenatal risk factors. However, existing research has largely been undertaken in developed countries. The current study explored the associations between receptive language and contextual, maternal and prenatal factors with a nationally representative sample of Chilean children aged between 36 and 48 months old, and then explored the stability of these associations two years later. Overall, the findings support other international longitudinal cohort studies regarding significant predictors of receptive language, emphasising that accesses to resources, maternal educational level with IQ outcomes, adolescent pregnancy, attending recommended medical appointments at pregnancy and being accompanied at childbirth by a significant person are significant factors to predict receptive language when children were aged between 36 and 48 months old; although at 60–72 months most predictors other than maternal education are supplanted by children’s previous receptive language scores. These findings suggest targets for early childhood policy development in Latin American in general and Chile in particular.

## Electronic supplementary material

Below is the link to the electronic supplementary material.

Electronic supplementary material 1 (DOCX 17 kb)

Electronic supplementary material 2 (DOCX 17 kb)
