# Supplementary material for: The Impact of Contextual, Maternal and Prenatal Factors on Receptive Language in a Chilean Longitudinal Birth Cohort
Source: Child Psychiatry Hum Dev. 2020 Nov 1;52(6):1106–17. doi: 10.1007/s10578-020-01091-5 (PMC8528774; doi:10.1007/s10578-020-01091-5)
Supplement: Supplementary file 1 — Electronic supplementary material 1 (DOCX 17 kb) [file 10578_2020_1091_MOESM1_ESM.docx]

# Table 1S

# *Attrition analyses between the entire cohort and the sample of this study*

|  | Entire Cohort  N = 15,175 (%) | Study’s sample  N = 3,921 (%) | *p* |
| --- | --- | --- | --- |
| Area of residence |  |  | ≤ .001 |
| Urban | 13,672 (90.1%) | 3,503 (89,3%) |  |
| Rural | 1,503 (9.9%) | 418 (10.7%) |  |
| Health provisional system |  |  | *ns* |
| Public system | 13,267 (89.4%) | 3,446 (89.7%) |  |
| Private system | 1,574 (10.6%) | 397 (10.3%) |  |
| Maternal educational level |  |  | *ns* |
| No formal education | 59 (0.4%) | 17 (0.4%) |  |
| Primary complete | 2,627 (17.3%) | 704 (18.1%) |  |
| Secondary complete | 5,944 (39.2%) | 1,578 (40.6%) |  |
| Vocational training | 4,613 (30.4%) | 1,163 (30.0%) |  |
| University studies | 1,661 (10.9%) | 398 (10.2%) |  |
| Postgraduate studies | 127 (0.8%) | 23 (0.6%) |  |
| Maternal IQ |  |  |  |
| WAIS Digit span subtest |  |  | *ns* |
| Below average | 9,366 (61.7%) | 2,623 (66.9%) |  |
| Average or high | 5,809 (38.3%) | 1,298 (33.1%) |  |
| WAIS Vocabulary subtest |  |  | *ns* |
| Below average | 4,912 (32.4%) | 1,352 (34.5%) |  |
| Average or high | 10,263 (67.6%) | 2,569 (65.5%) |  |
| Adolescent pregnancy |  |  | *ns* |
| Yes | 2,948 (19.7%) | 812 (20.7%) |  |
| No | 12,503 (80.3%) | 3,109 (79.3%) |  |
| Prenatal depression |  |  | *ns* |
| Yes | 1,788 (12.2%) | 377 (9.9%) |  |
| No | 12,822 (87.8%) | 3,441 (90.1%) |  |
| Smoking cigarettes at pregnancy |  |  | *ns* |
| Yes | 1,515 (10.1%) | 364 (9.3%) |  |
| No | 13,479 (89.9%) | 3,554 (90.7%) |  |
| Med. Appointments pregnancy |  |  | *ns* |
| Below recomm. | 1,970 (13.3%) | 490 (12.6%) |  |
| According recomm. | 12,843 (86.7%) | 3,389 (87.4%) |  |
| Mother accompanied at childbirth |  |  | *ns* |
| Yes | 11,538 (77%) | 2,831 (72.3%) |  |
| No | 3,437 (23%) | 1,085 (27.7%) |  |
| Postnatal depression |  |  | *ns* |
| Yes | 2,021 (13.5%) | 416 (10.7%) |  |
| No | 12,899 (86.5%) | 3,468 (89.3%) |  |
