# Supplementary material for: The Impact of Contextual, Maternal and Prenatal Factors on Receptive Language in a Chilean Longitudinal Birth Cohort
Source: Child Psychiatry Hum Dev. 2020 Nov 1;52(6):1106–17. doi: 10.1007/s10578-020-01091-5 (PMC8528774; doi:10.1007/s10578-020-01091-5)
Supplement: Supplementary file 2 — Electronic supplementary material 2 (DOCX 17 kb) [file 10578_2020_1091_MOESM2_ESM.docx]

# Table 2S

# *Attrition analyses between the sample that completed both language assessment, and the missing sample for the second assessment*

|  | Language measurement in both timepoints  N = 3,100 (%) | Language measurement in timepoint 1  N = 821 (%) | *p* |
| --- | --- | --- | --- |
| Area of residence |  |  | *ns* |
| Urban | 2,754 (88.8%) | 749 (91.2%) |  |
| Rural | 346 (11.2%) | 72 (8.8%) |  |
| Health provisional system |  |  | *ns* |
| Public system | 2,749 (90.5%) | 697 (86.7%) |  |
| Private system | 290 (9.5%) | 107 (13.3%) |  |
| Maternal educational level |  |  | *ns* |
| No formal education | 14 (0.5%) | 3 (0.4%) |  |
| Primary complete | 570 (18.6%) | 134 (16.5%) |  |
| Secondary complete | 1,271 (41.4%) | 307 (37.8%) |  |
| Vocational training | 911 (29.7%) | 252 (31.0%) |  |
| University studies | 288 (9.4%) | 110 (13.5%) |  |
| Postgraduate studies | 17 (0.6%) | 6 (0.7%) |  |
| Maternal IQ |  |  |  |
| WAIS Digit span subtest |  |  | *ns* |
| Below average | 2,110 (68.1%) | 513 (62.5%) |  |
| Average or high | 990 (31.9%) | 308 (37.5%) |  |
| WAIS Vocabulary subtest |  |  | *ns* |
| Below average | 1,094 (35.3%) | 258 (31.4%) |  |
| Average or high | 2,006 (64.7%) | 563 (68.6%) |  |
| Adolescent pregnancy |  |  | *ns* |
| Yes | 623 (20.1%) | 189 (23.0%) |  |
| No | 2,477 (79.9%) | 632 (77.0%) |  |
| Prenatal depression |  |  | *ns* |
| Yes | 283 (9.4%) | 94 (11.8%) |  |
| No | 2,735 (90.6%) | 706 (88.3%) |  |
| Smoking cigarettes at pregnancy |  |  | *ns* |
| Yes | 281 (9.1%) | 83 (10.1%) |  |
| No | 2,816 (90.9%) | 738 (89.9%) |  |
| Med. Appointments pregnancy |  |  | *ns* |
| Below recomm. | 384 (12.5%) | 106 (13.1%) |  |
| According recomm. | 2,687 (87.5%) | 702 (86.9%) |  |
| Mother accompanied at childbirth |  |  | *ns* |
| Yes | 2,207 (71.3%) | 624 (76.2%) |  |
| No | 890 (28.7%) | 195 (23.8%) |  |
| Postnatal depression |  |  | *ns* |
| Yes | 316 (10.3%) | 100 (12.3%) |  |
| No | 2,753 (89.7%) | 715 (87.7%) |  |
